# Switching from glargine+insulin aspart to glargine+insulin aspart 30 before breakfast combined with exercise after dinner and dividing meals for the treatment of type 2 diabetes patients with poor glucose control – a prospective cohort study

**DOI:** 10.1186/s12902-018-0297-4

**Published:** 2018-10-01

**Authors:** Jing Li, Liming Wang, Fen Chen, Dongxia Xia, Lingling Miao

**Affiliations:** Department of Endocrinology, The Affiliated Hospital of Medical School of Ningbo University, Zhejiang, China

**Keywords:** Glargine, Insulin aspart 30, Type 2 diabetes, Glycemic control

## Abstract

**Background:**

This study aimed to examine the switch from glargine+once daily insulin aspart (1 + 1 regimen) to glargine+insulin aspart 30 before breakfast combined with exercise and in patients with type 2 diabetes mellitus (T2DM) with poorly controlled blood glucose levels.

**Methods:**

Consecutive patients with poorly controlled T2DM (*n* = 182) were switched from the 1 + 1 regimen to glargine+insulin aspart 30 before breakfast in combination with exercise after dinner and dividing meals in two (same final calories intake). The insulin doses were adjusted according to blood glucose levels within 4 weeks after the switch and maintained for 12 weeks. Fasting blood glucose (FBG), 2-hpostprandial glucose (2hPG), glycosylated hemoglobin (HbA1c), body mass index (BMI), daily insulin dose, and hypoglycemia events were assessed.

**Results:**

Sixteen weeks after the switch, 2 h PG levels and HbA1c levels (from 8.5 to 7.4%, *P* = 0.001) were improved. The proportions of patients reaching the HbA1c targets of 7.5% were improved (from 22.5 to 58.7%, *P* = 0.001). Among the 182 patients, 24 (13.2%) divided one meal into two meals, and 23 (12.6%) divided two meals into four meals. Among all patients, 8.5% had to reuse insulin aspart before dinner after the study. One patient with diarrhea and poor appetite experienced severe hypoglycemia. The rate of hypoglycemia was 3.76 events/patient-year. The daily insulin Aspart 30 dose was higher than the original insulin aspart dose (*P* = 0.001).

**Conclusions:**

For patients with poorly controlled T2DM under the 1 + 1 regimen, switching to glargine+insulin aspart 30 before breakfast combined with exercise after dinner and dividing meals showed promising benefits.

## Background

Type 2 diabetes (T2DM) is an endocrine disorder characterized by hyperglycemia that results from variable degrees of insulin resistance and deficiency [1]. Chronic hyperglycemia associated with T2DM eventually leads to renal, neurologic, and cardiovascular complications [1]. In 2014, the worldwide prevalence of T2DM was 9% in men and 7.9% in women [2]. In China, a cross-sectional study carried out in 2010 showed an estimated prevalence of T2DM of 12.1% in men and 11% in women, and a 50.1% prevalence of prediabetes [3]. Therefore, T2DM represents a serious threat to the health of the Chinese population and this state should become worse because of the Westernization of the nutritional patterns in China [4].

For patients with a long course of T2DM that is still uncontrolled despite glargine combined with oral drugs, a regimen of glargine+prandial insulin is usually proposed [5–7]. The “1 + 1 regimen” (glargline+ 1-time prandial insulin) is usually started first, but it often has to be changed to the “1 + 3 regimen” (glargine+ 3-times prandial insulin) to achieve glycemic control [5–8]. With time, β-cell function progressively declines and secretagogues gradually lose their effectiveness, ultimately leading to the progression from the 1 + 1 to the 1 + 3 regimen [5–7]. The 1 + 3 regimen is associated with better glycemic control than the 1 + 1 regimen [8–10].

The 1 + 3 regimen requires injections before meals every day, resulting in low compliance. Shifting from the 1 + 1 regimen to the 1 + 3 regimen is associated with significantly decreased quality of life and treatment adherence [11]. The APOLLO trial showed that a single daily injection of insulin glargine resulted in better quality of life and compliance than injections thrice daily [10].

We hypothesized that optimizing treatment approaches based on a single daily injection could achieve appropriate glycemic control, possibly because of better compliance, compared with three injections each day. Therefore, this study aimed to examine the switch from glargine+once daily insulin aspart 30 (1 + 1 regimen) to glargine+insulin aspart 30 before breakfast combined with exercise after dinner and reduced servings in patients with type 2 diabetes mellitus (T2DM) with poorly controlled blood glucose levels, and to assess whether targets of the blood glucose and HbA1c were achieved.

## Methods

### Study design and subjects

This was an observational prospective single-arm cohort study (registration number: ChiCTR-OOC-17011177). Consecutive patients with T2DM treated at the Endocrinology Department of the Affiliated Hospital of Medical School of Ningbo University during June 2014 and February 2015 were consecutively enrolled and followed. This study was approved by the ethics committee of the Affiliated Hospital of School of Medicine of Ningbo University. Informed consent was obtained from all patients.

The inclusion criteria were: 1) Chinese patients with type 2 diabetes according to the 1999 criteria of the World Health Organization [12]; 2) 50–82 years of age; 3) T2DM course of 10–30 years; 4) had constantly received the 1 + 1 regimen (combined with at least two kinds of oral antidiabetic drugs including secretagogues, α-glycosidase inhibitor, metformin, TZD, and DPP-IV inhibitors) for at least 3 months; 5) HbA1c > 6.5% but < 11.0%; 6) fasting blood glucose (FBG) of 5–7 mmol/l and at least one postprandial blood glucose (PG) > 11.0 mmol/l; and 7) patients were willing and able to use a blood glucose meter, and to provide informed consent. The exclusion criteria were: 1) non-type 2 diabetes patients (such as type 1 diabetes, diabetes secondary to pancreatic diseases, or diabetes due to drugs or chemicals); 2) pregnant or lactating women, or women willing to be pregnant; 3) patients accompanied with acute diabetic complications such as diabetic ketoacidosis and hyperosmolar coma; 4) patients with liver and renal failure (alanine transaminase > 2 times the upper limit of normal or patients with obvious kidney diseases or serum creatinine > 133 μmol/L); 5) other endocrine and metabolic diseases, newly discovered tumors, or autoimmune diseases; 6) diseases in the thyroid, pituitary, or adrenal gland, or any other endocrine disease; 7) participating or had participated in another study within 3 months; or 8) received steroids within 3 months. In addition, the patients showing one of the following criteria were ruled out of the study: 1) allergic reactions, severe adverse reactions, or intolerance after receiving insulin aspart 30, acarbose, or metformin; 2) any protocol deviation; or 3) could not continue this study due to any factors, for which the reasons would be recorded.

### Intervention

Prior to the study, all patients received guidelines on diet and exercise [13, 14], as well as on the use of blood glucose meters, on self-monitoring knowledge, and on hypoglycemia symptoms and appropriate countermeasures.

Each patient visited the outpatient 10 times during the 16-week study treatment period. During the first 4 weeks (adjustment period), each patient was seen each week, and then every 2 weeks for weeks 5–16.

#### Adjustment period (1–4 weeks)

The patients were required to perform moderate-intensity exercise for > 30 min after dinner each day (e.g., walking, jogging, square dancing, ball games). Moderate-intensity exercise was defined as keeping the exercise workload at 65% of maximum heart rate (HR max = 220 - age). This method was used because it can be easily self-monitored. During this period, patients were asked to monitor fasting blood glucose and 2-h postprandial glucose (2hPG) after each meal, every 3 days. Then, they adjusted their antidiabetic drugs according to the results.

**Fig. 1:**
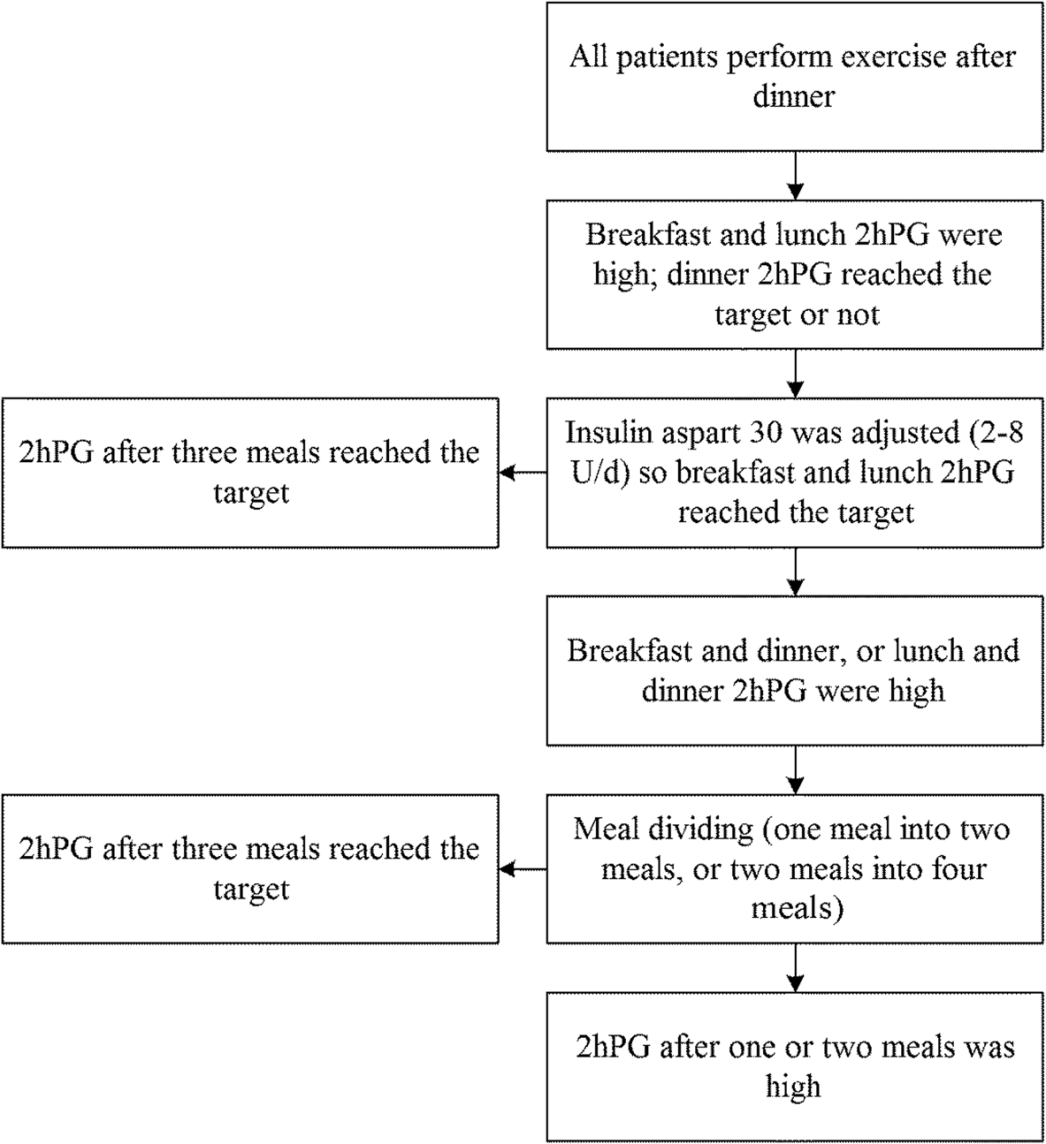
Meal adjustment algorithm in patients receiving glargine+premixed insulin before breakfast. All the patients were required to exercise 30 min (moderate intensity) after dinner

The original 1 + 1 regimen was discontinued and breakfast insulin aspart 30 was added to glargine. In addition, an individualized treatment approach was developed according to the patients’ body mass index (BMI). The adjustment procedures were: 1) the initial dose of insulin aspart 30 analogues was set at 1.5 times the dose of breakfast insulin aspart in the original “1 + 1” regimen or as 6 U. Then, the insulin aspart 30 was gradually adjusted by 2–8 U until at least one of the 2 h PG level readings after breakfast or lunch met the targets; 2) dividing one meal into two meals was required in the following situations: a) 2 h PG failing to meet the target after one or two meals (except for patients with 2 h PG failing to meet the requirement at both breakfast and lunch); or b) hypoglycemia between meals. 3) If the 2 h PG levels still failed to meet the standard after dividing meals, then the patients were switched to glargine+multiple prandial insulin doses after the study (Fig. 1). In terms of dividing meals, the patients were asked to reduce by 1/4–1/3 (about 25–50 g) their carbohydrate-rich food amount (rice, dried pasta, or bread) for the meal which was supplemented with equal amounts of low-fat crackers, bread, or other carbohydrates at 2 h after the meal. If their BMI failed to fall below 25 kg/m^2^, the snack at 2 h after meal was withdrawn in order to control the weight and to improve insulin sensitivity of overweight patients.

In addition, patients were given oral administration of acarbose at dinner (minimum and maximum doses of 50 mg qd and 100 mg tid) and metformin (minimum and maximum doses of 0.5 g bid and 1.0 g bid). At the same time, they stopped taking secretagogues, TZDs, and DPP-IV inhibitors. The doses of insulin and oral drugs were adjusted according to the blood profiles. Patients were asked to have regular meal times. The purpose of the treatment was to achieve 2 h PG < 11.0 mmol/L and the target HbA1c level was set to 7.5%.

#### Stable period (5–16 weeks)

Once adjusted, the treatment protocol was maintained for 12 weeks, and had to remain unchanged. Nevertheless, if fine adjustments were needed due to disease conditions, the dose of glargine and variety of oral hypoglycemic agents (metformin tablet and acarbose tablet) could only be reduced rather than increased, while the doses of insulin aspart 30 could be increased or decreased.

### Data collection

The following baseline data were recorded: age, gender, course of disease, insulin dose, BMI, HbA1c, fasting glucose, glucose before and 2 h after meals, glucose before sleep and insulin injection time-point before main meal (breakfast, lunch, or dinner). At the 16th weekend, HbA1c was measured.

### Study drugs

In this study, insulin glargine (Lantus) (Sanofi Aventis, Paris, France) and insulin aspart 30 (NovoMix 30 Flexpen) (Novo Nordisk, Bagsvaerd, Denmark) were selected, both of which were injected simultaneously and separately on both sides around the umbilicus before breakfast. Other drugs included metformin tablet (Glucophage) (Bristol-Meyer Squibb, New York, NY, USA) and 100 mg Glucobay (acarbose tablet) (Bayer HealthCare Pharmaceuticals, Montville, NJ, USA).

### Laboratory parameters

All blood glucose measurements were performed using fingertip blood using a steady blood glucose meter (Johnson & Johnson, New Brunswick, NJ, USA). The average blood profiles 1 week prior to participation and at the 16th week of treatment (average value within 1 week was taken) were monitored. HbA1c was monitored using high-pressure liquid chromatography (Bio-Rad, Hercules, CA, USA).

### Outcomes

The primary outcomes were changes in HbA1c and 2 h PG at the end of the study compared to the baseline levels. The secondary outcomes were: 1) achieving HbA1c < 7.5%; and 2) changes of insulin dose and BMI.

### Adverse effects

The number of hypoglycemia events and their duration and severity throughout the study period were analyzed. Hypoglycemia was defined as the patient feeling or were found to have symptoms and signs of hypoglycemia, and blood glucose < 3.9 mmol/l at any time point. Severe hypoglycemia referred to the occurrence of coma, psychotic symptoms, or necessity of injection of glucose, the remaining cases were considered as general hypoglycemia. If patients showed recurrent general hypoglycemia within a few days, they were asked to inform their doctors by telephone. If non-drug factors (such as excessively less or late ingress, or excessive exercise, etc.) were excluded from causing hypoglycemia in the patients, the insulin dose had to be adjusted promptly. Patients suffering from severe hypoglycemia had to visit the hospital for treatment immediately. All other adverse reactions were recorded.

### Statistical analysis

Categorical variables were expressed as frequency and percentage, while continuous variables were expressed as mean, standard deviation, median, maximum and minimum, as appropriate. Changes between data before and after treatment were compared using analysis of covariance (ANCOVA). Categorical variables were described using frequency, and changes between data before and after treatment were analyzed using the chi-square test or non-parametric tests. Changes of HbA1c levels were assessed using the hybrid model. Statistical analyses were performed using SAS 6.12 software (SAS Institute, Cary, NY, USA). Two-sided *P*-values < 0.05 were considered statistically significant.

## Results

### Characteristics of the patients

Two hundred and two patients were screened. Five patients were reluctant to participate since they could not adhere to the exercise program. Three patients were lost to follow-up. Twelve patients withdrew during treatment: three because they were unable to meet the requirements of the exercise program and nine because of adverse reaction to the oral hypoglycemic drugs. Ultimately, 182 patients were analyzed. Table 1 presents the baseline characteristics of the patients. The BMI of 87 (47.8%) patients was below 25 kg/m^2^.

**Table 1:** Baseline characteristics of the patients

Variables	*n* = 182
Gender	
Male (n, %)	90 (49.5)
Female (n, %)	92 (50.5)
Age (years)	62.0 ± 10.9
BMI (kg/m^2^)	24.9 ± 2.7
Course of disease (years) (min, max)	14.6 ± 4.5 (10, 21)
Insulin glargine dose (U)	25.5 ± 8.2
Daily insulin dose (U)	32.2 ± 8.5

*BMI* body mass index

### Glucose and HbA1c levels

Table 2 present the FBG and PG profiles before and after switching to glargine+insulin aspart 30 before breakfast. Switching to glargine+insulin aspart 30 before breakfast resulted in lower breakfast, lunch, and dinner 2 h PG and HbA1c, (all *P* = 0.001).

**Table 2:** Comparisons of blood glucose profile before and after glargine+ Insulin Aspart 30 before breakfast

Variables	Glargine+once daily insulin aspart	Glargine+insulin aspart 30 before breakfast	*P*
FPG (mmol/L)	6.3 ± 0.6	6.2 ± 0.5	0.085
Breakfast 2 h PG (mmol/L)	13.9 ± 2.8	9.8 ± 2.6	0.001
Before lunch	9.1 ± 2.2	6.3 ± 1.9	0.001
Lunch 2 h PG (mmol/L)	13.8 ± 2.8	9.5 ± 2.5	0.001
Before dinner	8.7 ± 2.6	6.5 ± 2.1	0.001
Dinner 2 h PG (mmol/L)	12.3 ± 2.9	9.6 ± 2.5	0.001
Before sleep	8.5 ± 2.0	7.3 ± 1.8	0.001
HbA1c (n, %)	8.5 ± 1.6	7.4 ± 1.2	0.001
Rate of reaching HbA1c < 7.5% (n, %)	41 (22.5)	107 (58.7)	0.001

*FPG* fasting plasma glucose, *2 h PG* 2-h postprandial glucose

*n* = 182

Changes between data before and after treatment were compared using analysis of covariance (ANCOVA)

Frequencies were analyzed using the chi-square test

**Fig. 2:**
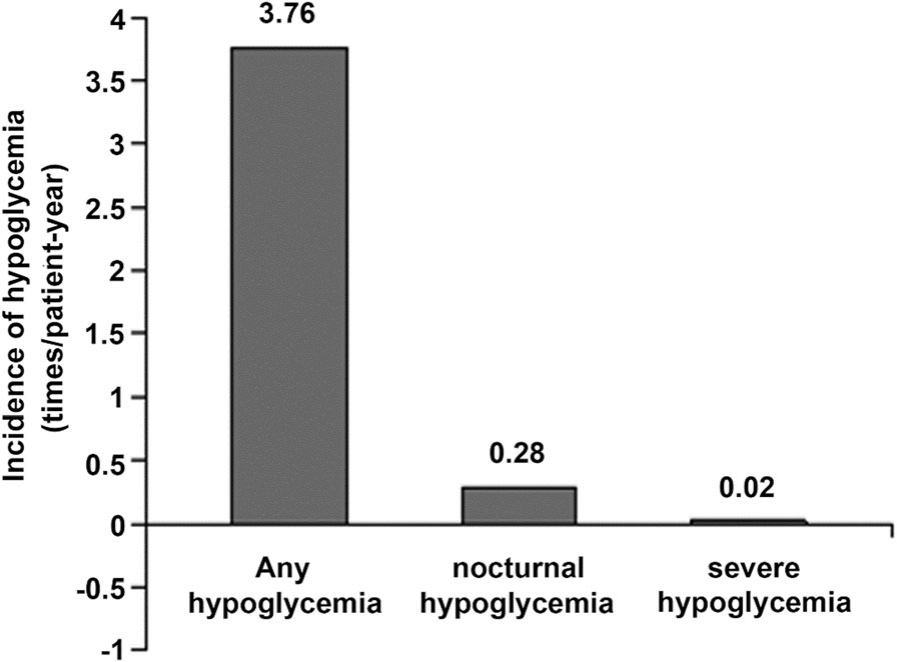
Incidence of hypoglycemia (times/patient-year) after switching to glargine+premixed insulin before breakfast. *n* = 182

Tables 2 show the HbA1c levels before and after switching to glargine+insulin aspart 30 before breakfast. In addition, the HbA1c targets < 7.5% were examined. Sixteen weeks after the switch, 2 h PG levels at each meal and HbA1c levels were improved (all *P* = 0.001). The proportions of patients reaching the HbA1c targets of 7.5% were improved (7.5%: from 22.5 to 58.7%, *P* = 0.001); Table 3 and Fig. 2 present the hypoglycemia events, BMI, and insulin doses. The daily insulin dose was higher after the switch (*P* = 0.001). Patients had lost weight 16 weeks after the switch (*P* = 0.002). One patient suffered from severe hypoglycemia. The rate of hypoglycemia was 3.76 events/patient-year. The daily insulin aspart 30 dose was higher than the original insulin aspart dose (*P* = 0.001).

**Table 3:** Incidence of hypoglycemia (times/patient-year), insulin dose (U), and BMI (kg/m^2^) after switching to glargine+ insulin aspart 30 before breakfast

Item	Glargine+once daily insulin aspart	Glargine+insulin aspart 30 before breakfast	P
Insulin glargine dose	25.5 ± 8.2	24.8 ± 8.0	0.410
Daily insulin dose	32.2 ± 8.5	40..7 ± 8.1	0.001
BMI	24.9 ± 2.7	24.1 ± 2.2	0.002
Patients with hypoglycemia event	N/A	159/182 (87.3%)	N/A
Hypoglycemia (times/patient-year)	N/A	3.76	N/A

*BMI* body mass index, *N/A* not available

Results are presented as mean ± SD

*n* = 182

Changes between data before and after treatment were compared using analysis of covariance (ANCOVA)

### Insulin dose

Table 4 presents the proportions of patients receiving or needing prandial insulin at different meals before the switch and patients still needing prandial insulin at the end of study, The proportions of patients receiving prandial insulin at meals were 60.4% at breakfast, 23.1% at lunch, and 16.9% at dinner. Before the switch. The proportions of patients needing prandial insulin at meals were, 96.1% at breakfast, 86.8% at lunch, and 30.2% at dinner. Sixteen weeks after the switch, the proportion of patients needing prandial insulin at dinner was decreased to 8.5% by dividing out dinner into two meals and exercise after dinner.

**Table 4:** Proportions of patients receiving or needing additional prandial insulin at different meal time before and after switching to glargine+insulin aspart 30 before breakfast

	Receiving prandial insulin before switching to glargine+insulin aspart 30	Needing prandial insulin before switching to glargine+insulin aspart 30	Needing prandial insulin at dinner after switching to glargine+insulin aspart 30
Breakfast insulin aspart (n, %)	110 (60.4)	175 (96.1)	48 (26.3)
Lunch insulin aspart (n, %)	42 (23.1)	158 (86.8)	38 (20.8)
Diner insulin aspart (n, %)	30 (16.9)	55 (30.2)	16 (8.5)^a^

^a^Proportions of patients needing insulin at dinner 16 weeks after switching to glargine + insulin aspart 30

*n* = 182

Frequencies were analyzed using the chi-square test

### Meal adjustment

**Fig. 3:**
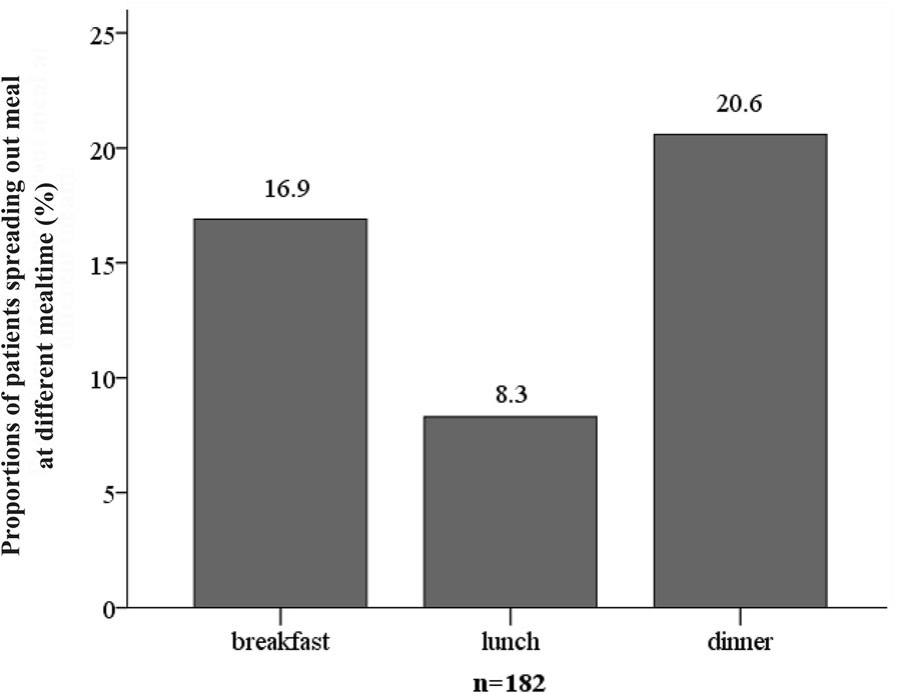
Proportions of patients needing to reduce their meals at different after switching to glargine+premixed insulin. *n* = 182

Meal adjustments are shown in Fig. 3. At the end of study, the ratio of patients dividing meal(s) at the 16th week after switching was 16.9% for breakfast, 8.3% for lunch, and 20.6% for dinner. Dividing at least one meal into two meals was conducted by 47 (25.8%) patients, among whom 23 (12.6%) patients finally needed dividing two meals into four meals, and the remaining 24 (13.2%) patients needed dividing one meal into two meals.

## Discussion

This study aimed to examine the switch from glargine+once daily insulin aspart (1 + 1 regimen) to glargine+insulin aspart 30 before breakfast combined with exercise after dinner and dividing meals in patients with type 2 diabetes mellitus (T2DM) with poorly controlled blood glucose levels, and to assess whether the blood glucose targets were achieved after dinner. The results suggest that for patients with poorly controlled T2DM under the 1 + 1 regimen, switching to glargine+insulin aspart 30 after breakfast could have promising benefits.

In this study, we adopted a new regimen of glargine+insulin aspart 30 before breakfast, which meant to separately inject glargine and insulin aspart 30 before breakfast, hereby to make the 30% of insulin aspart to play the action of insulin before breakfast. At lunch, the remaining 70% of protamine insulin aspart started to peak, and played the role of lunch insulin. Therefore, the principle of this regimen is probably similar to that of the 1 + 2 regimen. Nevertheless, since insulin aspart 30 has a fixed proportion, its regulatory effects on blood glucose after breakfast and lunch may not be as flexible as that of the 1 + 2 and 1 + 3 regimens. Thus, to compensate this disadvantage, patients were asked to divide one or two meals to improve the blood glucose fluctuations after meal. Therefore, 47 (25.8%) of the 182 patients had to divide their at least one meal,into two meals, of which it was respectively adopted in 16.9% and 8.3% of the 182 patients at breakfast and lunch.

The glargine+insulin aspart 30 before breakfast regimen did not affect 2 h PG after dinner. Indeed, the proportion of patients who needed insulin injection before dinner was not high before switch (30.2%). Patients from western countries often consume most of their energy of the day at dinner, while older Chinese patients often have a lower calorie intake at dinner. Thus, the need for insulin injection before dinner was not high. This phenomenon was also confirmed in a study by Feng et al. [15], in which the 1 + 1 regimen showed the highest proportion of injections before breakfast (55.9%) and the lowest proportion of injections before dinner (20.3%). This suggests that the need for insulin before dinner could be less in Chinese elderly patients with T2DM. Secondly, acarbose, an α-glucosidase inhibitor, played a synergistic hypoglycemic effect with glargine at dinner, which could have reduced prandial insulin. Thirdly, 30 min of exercise after dinner and/or dividing dinner into two meals were adopted. Compared to baseline, the exercise and/or dividing dinner could lower the proportion of patients who needed insulin at dinner from 30.2 to 8.5%.

Exercise efficiently improves the postprandial blood glucose levels of patients with T2DM [1–3, 14]. The insulin-like effect generated by exercise can significantly decrease the blood glucose levels, especially for patients with seriously impaired glucose metabolism [13]. According to the present study, the increased area under the glucose curve among the patients who performed postprandial exercises declined more obviously than that of patients who just walked once a day, especially for the patients who performed exercise after dinner. In addition, this effect on postprandial blood glucose was more obvious for patients who consumed a large amount of carbohydrates [16]. Chinese patients with T2DM have both these features, i.e. using carbohydrates as main calorie source and exercising after dinner.

It is worth mentioning that the long disease course in the present study may have resulted in a poor effect of secretagogues. Nevertheless, because of the high proportion of carbohydrates in the Chinese diet compared to the Western diet [17], the therapeutic effect of α-glucosidase inhibitor is significantly superior to that in European diabetic population [18, 19]. Meanwhile, acarbose plays a significant “peak shaving” effect through delaying the absorption of carbohydrates, thereby saving the prandial insulin dose to some extent. Therefore, patients were asked to stop secretagogues. Instead, they took at least 50 mg of acarbose at dinner or, if possible, at the three meals.

In this study, for patients with poorly controlled T2DM under the 1 + 1 regimen, switching to glargine+insulin aspart 30 before breakfast showed promising benefits. A previous study showed that basal-bolus insulin detemir/insulin aspart could be more effective than biphasic insulin aspart in reducing HbA1c [20]. Another study showed that a flexible glargine dosing regimen achieved better outcomes than a fixed one [21]. A systematic review showed that regimens based on prandial premixed insulin analogues (thus providing both basal and prandial insulin coverage) could result in better overall, preprandial, and postprandial glycemic control compared to basal insulin alone regimens [22]. Nevertheless, direct comparisons among studies are difficult because of the variety of regimens.

In this study, the overall hypoglycemia and nocturnal hypoglycemia rates were not very high, suggesting that this approach could be acceptable. Compared to conventional multiple preprandial insulin regimens, injecting premixed fixed proportion of insulin before breakfast may increase the risk of hypoglycemia between meals, but this shortcoming can be solved using dividing one meal into two meals. The included patients were mainly elderly patients with a long T2DM course. These patients often display poor self-regulation ability of blood glucose levels and higher risk of hypoglycemia [23, 24]. Therefore, the safety of treatments is particularly important in this population,since hypoglycemia often leads to even more serious consequences in these patients, such as aggravating cardiovascular diseases and neurological disorders, fall fracture, coma, and even death [23, 24]. Based on these risks, the target HbA1c level was set to 7.5% in this study.

According to previous studies, the proportion of Chinese elderly T2DM patients doing exercise is continuously rising [25, 26]. In this study, patients were provided with health guidance and individualized exercise programs according to their living habits and hobbies, which resulted in good exercise compliance in the majority of patients. It is noteworthy that the subjects in this study were middle-aged and elderly patients (50–82 years old) and most of them had regular daytime activities (housework, grocery shopping, taking care of the third generation of children, etc.). Thus, it could be difficult for them to improve blood glucose after breakfast and lunch by performing additional exercise. Meanwhile, nowadays in China, nocturnal exercise like night “square dance”, night walking, cycling, jogging or boxing, etc., are increasingly popular. Therefore, postprandial exercise after dinner was conform to the life habits of most middle-age and elderly patients with T2DM.

In this study, the overweight patients were asked to reduce carbohydrate-rich food intake by about 25–50 g in the meal for which 2hPG levels failed to meet the targets. Therefore, the weight of the overweight patient could be decreased by properly reducing their food intake and increasing their exercise (regular moderate-intensity exercise for 30 min after supper).

Our success in terminating insulin before dinner without effect on postprandial blood glucose is possibly based on the following factors. First, we and the Tianjin ‘1 + 1’ regimen study [15] both found that the proportion of patients using insulin at dinner was low (30.2% and 20.3%, respectively). Therefore, dinnertime insulin was not widely used in these patients. Secondly, 2 h PG levels can be further improved through regular moderate-intensity exercise for 30 min after dinner and dividing dinner into two meals [27, 28]. Thirdly, acarbose has a synergistic effect on insulin glargine. In the present study, only 8.5% of the patients with T2DM needed dinnertime insulin injection to control 2 h PG by using glargine+insulin aspart 30 in combination with exercise after dinner and dividing dinner.

The present study is not without limitations. The sample size was small and from a single center. No control group was neither included, nor other types of insulin regimens or dosages were tried. Only Chinese middle-aged and elderly subjects were recruited, limiting the generalizability of the results. We selected patients of > 50 years of age because they were retired or nearly retired and they could more easily keep up with the 30-min of exercise compared with younger working people, probably with children at home, and with less available time. Since it was an observational study, a run-in period was not included and we could not reliably record the hypoglycemic events before switching. Additional studies are necessary to compare the efficacy and safety of this new insulin regimen with other regimens.

## Conclusions

In conclusion, for patients with poorly controlled T2DM under the 1 + 1 regimen, switching to glargine+ insulin aspart 30 before breakfast combined with exercise after dinner and dividing meals showed promising benefits. Interventional study was needed to evaluate efficacy and safety.

## Abbreviations

2hPG: 2-hpostprandial glucose
ANCOVA: Analysis of covariance
FBG: Fasting blood glucose
HbA1c: Glycosylated hemoglobin
T2DM: Type 2 diabetes mellitus
